# Operationalizing Cooperative Research for Infectious Disease Surveillance: Lessons Learned and Ways Forward

**DOI:** 10.3389/fpubh.2021.659695

**Published:** 2021-09-10

**Authors:** Kenneth B. Yeh, Falgunee K. Parekh, Kairat Tabynov, Kaissar Tabynov, Roger Hewson, Jeanne M. Fair, Sandra Essbauer, John Hay

**Affiliations:** ^1^MRIGlobal, Gaithersburg, MD, United States; ^2^EpiPointe, LLC, Cary, NC, United States; ^3^International Center for Vaccinology, Kazakh National Agrarian Research University, Almaty, Kazakhstan; ^4^Public Health England, Salisbury, United Kingdom; ^5^London School of Hygiene and Tropical Medicine, London, United Kingdom; ^6^Los Alamos National Laboratory, Los Alamos, NM, United States; ^7^Bundeswehr Institute for Microbiology, Munich, Germany; ^8^Jacobs School of Medicine and Biomedical Sciences, Buffalo, NY, United States

**Keywords:** cooperative research, global health security, infectious disease surveillance, capacity building, Central Asia, COVID-19

## Abstract

The current COVID-19 pandemic demonstrates the need for urgent and on-demand solutions to provide diagnostics, treatment and preventative measures for infectious disease outbreaks. Once solutions are developed, meeting capacities depends on the ability to mitigate technical, logistical and production issues. While it is difficult to predict the next outbreak, augmenting investments in preparedness, such as infectious disease surveillance, is far more effective than mustering last-minute response funds. Bringing research outputs into practice sooner rather than later is part of an agile approach to pivot and deliver solutions. Cooperative multi- country research programs, especially those funded by global biosecurity programs, develop capacity that can be applied to infectious disease surveillance and research that enhances detection, identification, and response to emerging and re-emerging pathogens with epidemic or pandemic potential. Moreover, these programs enhance trust building among partners, which is essential because setting expectation and commitment are required for successful research and training. Measuring research outputs, evaluating outcomes and justifying continual investments are essential but not straightforward. Lessons learned include those related to reducing biological threats and maturing capabilities for national laboratory diagnostics strategy and related health systems. Challenges, such as growing networks, promoting scientific transparency, data and material sharing, sustaining funds and developing research strategies remain to be fully resolved. Here, experiences from several programs highlight successful partnerships that provide ways forward to address the next outbreak.

## Background

**Figure 1:**
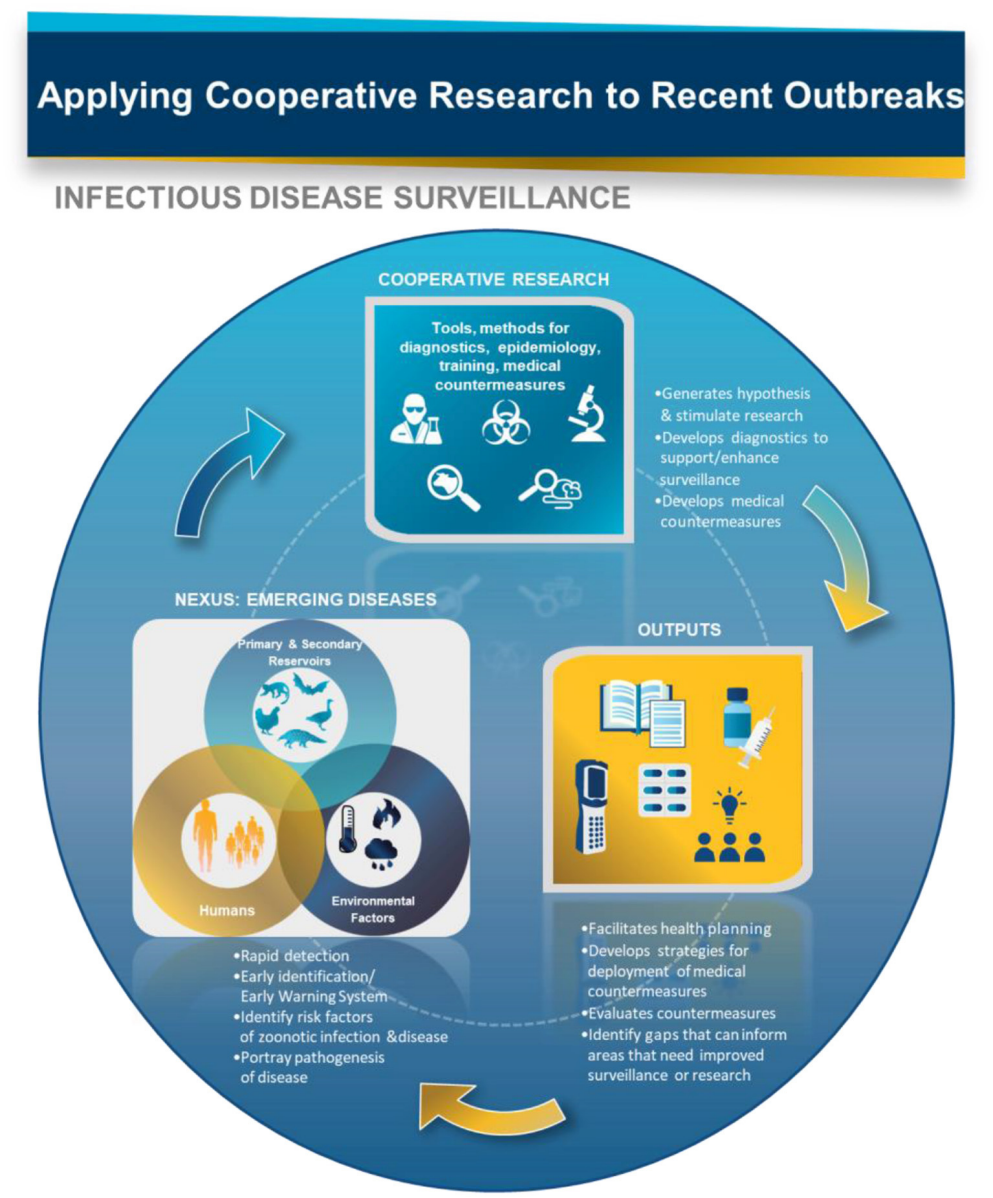
Infectious disease surveillance is a vital component in the response to disease outbreaks and the subsequent development of effective countermeasures. COVID-19 demonstrates the need for faster turnaround to meet technical, logistical, and production demands.

The COVID-19 pandemic exemplifies the importance of infectious disease surveillance in various aspects of preparedness, response, hypothesis generation for research purposes, implementation of interventions like mask-wearing and development of therapeutic and vaccine products. In addition to enabling early detection, the ongoing and systematic monitoring of infectious disease surveillance data allows us to assess the transmission dynamics of disease, which can then help develop predictive models with a higher level of accuracy. These predictive models, in turn, can help inform the development of preparedness and response policies that can then curb transmission. Moreover, effective infectious disease surveillance allows us to understand the clinical presentation of disease, the pathogen, the detection of the pathogen in natural foci, host risk factors associated with severity or protection, changes in these risk factors and which populations are most at risk. This type of information is critical to the development of effective interventions, prophylaxis, therapeutics and vaccines against infectious diseases (Figure 1). For example, if we are able to assess the reason behind asymptomatic or mild cases of disease, we may have a better understanding of the immune correlates of protection in those individuals, which then can inform effective vaccine development.

The COVID-19 pandemic has also created a global “all hands-on deck” effect where national governments recognize that they must collaborate internationally across sectors and among communities and individuals to achieve containment (1). Industry needs assistance to accelerate funding opportunities, as exemplified by the US Food and Drug Administration Emergency Use Authorization (FDA EUA), which was essential in efforts to make COVID diagnostic tests and treatments more available, by streamlining the regulatory process. Another important challenge is the ability to operationalize research outputs and increase success rates for products in the development pipeline. Well-known examples exist where opportunities arose during crises and those who collaborated effectively were better prepared to excel (2). Similarly, collaboration during an outbreak enhances communication and coordination, and the numerous resulting R&D outputs are additional beneficial by-products. While cooperative research can take place in many forms among public and private partnerships within a country, as well as in collaborations among different countries, we focus on those international programs aimed at biological threat reduction and enhancing biosecurity engagement (3, 4).

Cooperative research programs develop capacity that can be applied to infectious disease surveillance and research that enhances detection, identification, and response to emerging and re-emerging pathogens with epidemic or pandemic potential. In this paper, we describe work done in three Central Asia countries: Kazakhstan, Kyrgyzstan, and Tajikistan. It quickly became apparent that program funding from Germany, UK and US programs had overlapping research programs and activities. Recognizing that these similar efforts led to opportunities to reinforce cooperation, we describe research that has been operationalized in each country: Kazakhstan (Germany, UK, and US), Kyrgyzstan (UK, US, Canada, China, Russia and WHO), and Tajikistan (UK). Ideally, cooperative research continues peer mentorships that first promote international norms and best practices to encourage hypothesis-based studies and scientific transparency. Successful mentorships form collaborations that create greater networks for participants, furthering scientific knowledge, infrastructure and related capabilities. Other benefits include the return on relationships (e.g., joint publications) and construction of sustainable networks that arise as a product of collaboration (5).

Robust networks, based on the above approaches, offer agility, creativity, and trust which can accelerate engagements through familiarity and rapport among colleagues and peers. The elements of further multi-disciplinary and multi-sectoral collaborations are key to furthering outputs that can be operationalized. However, a major challenge is the often-different set of expectations among program funders and partner country recipients. Getting these aspects resolved is essential to building goodwill and trust, as well as promoting mutually-beneficial good practices in partner countries.

Our three case histories are from countries of the Former Soviet Union, which is a common area of interest for biosecurity-based engagement programs. These countries have histories of state programs for biological weapons development (6), thus appealing to agencies interested in countering biological weapons, preventing use and reducing threats, in line with the Geneva Convention of 1975. Our three examples illustrate outputs from cooperative research that point to its value in real-life public health situations.

## Cooperative Biological Research Programs in the Republic of Kazakhstan

**Figure 2:**
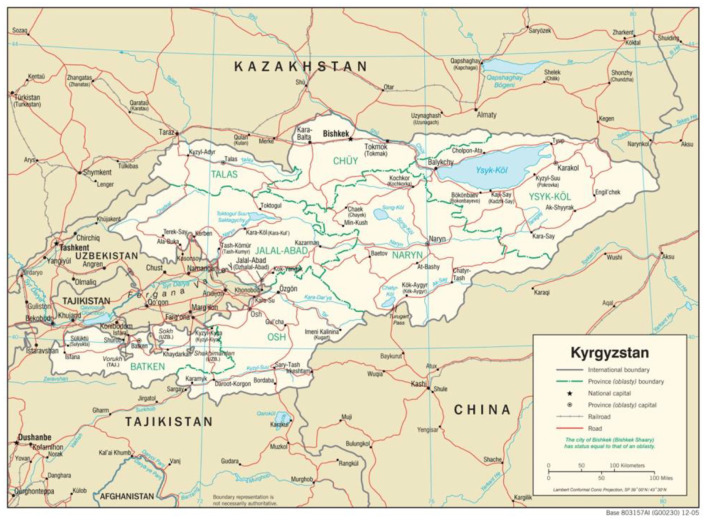
Political map of Kyrgyzstan, showing its proximity to Kazakhstan and Tajikistan. The major cities mentioned in the text are shown (Almaty, Bishkek, Osh and Dushanbe). Map courtesy of the University of Texas Libraries (lib.utexas.edu).

The US Defense Threat Reduction Agency (DTRA) has implemented its Biological Threat Reduction Program (BTRP) with Kazakhstan for over 20 years (4). DTRA funded the construction and commissioning in 2018 of the Central Reference Laboratory (CRL) in Almaty, Kazakhstan (Figure 2), which is now operational and serves as national level reference laboratory. During the CRL's commissioning, DTRA also supported several research studies intended to bridge activities that the CRL would eventually house. The CRL involves cooperation among three Kazakh ministries, DTRA and their collaborators which included scientists from the US and UK, and contractors who implemented much of the work. As a result of this activity, substantial scientific and medical progress has been made, both at the practical and personal level and Kazakh scientists who have taken part in DTRA programs are now publishing independent work in the international press (7).

The CRL has recently supported research by Kazakhstani scientists for COVID-19 research that includes animal biosafety laboratory studies for a national vaccine. Kazakh government, universities and commercial companies fund additional research.

The German Federal Foreign office in 2013 launched the German Biosecurity Program (GBP) in order to implement sustainable biosafety and biosecurity projects in various countries. The current program phase runs from 2020 until 2022 and is currently active in nine countries including two supraregional projects (8). Through the German Federal Foreign Office's German Biosecurity Program, the Bundeswehr Institute of Microbiology (BIM) and the Deutsche Gesellschaft für Internationale Zusammenarbeit GmbH (GIZ) have managed a project in Kazakhstan for the past seven years in collaboration with key Kazakh partners including the aforementioned NSCEDI at the CRL and the Research Institute for Biosafety Problems (RIBSP). Under the auspices of the G7 Global Partnership against the Spread of Weapons and Materials of Mass Destruction, the GBP focuses on surveillance, detection and diagnostics, biosafety and biosecurity including work published on Crimean Congo haemorrhagic fever, tick-borne encephalitis virus, *Rickettsia* and orthohantaviruses (9–12).

The GBP also finances a e-learning platform (German Online Platform for Biosecurity & Biosafety (GO4BSB), which contributes to development of a sustainable network in Kazakhstan which includes COVID-19 training and information, available in Russian language. The initiative is a collaborative effort by the Bernhard Nocht Institute for Tropical Medicine, BIM, Friedrich Loeffler Institut, Federal Research Institute for Animal Health, Robert Koch Institute and GIZ. Informal interactions among collaborators of the US and German cooperative programs in Kazakhstan also enhanced collaboration.

The cooperative research outputs, namely the CRL infrastructure, capacity building through workforce training, and the established multi-national collaboration and networks, have all been leveraged in response to the COVID-19 pandemic. Examples include publishing the genetic sequence of spike protein, development of a COVID-19 subunit vaccine, and preclinical testing of this subunit vaccine. Through the e-learning platform COVID-19 training and information, available in Russian language, was also deployed.

## COVID-19 Responses in the Kyrgyz Republic

For the past 15 years or so, Kyrgyzstan has been the recipient of multinational cooperative research assistance, aimed at resolution of health problems. Entities that have worked there include ISTC (International Science and Technology Committee; based in Moscow but funded by a consortium of countries), CRDF (Civilian Research and Development Foundation; US), Dstl (Defence science and technology laboratories; UK) and the Canadian Weapons Threat Reduction Program. As a result of these joint efforts, the Republic has acquired a substantially more developed health surveillance and treatment ability (13). COVID was first detected in Kyrgyzstan in March 2020, following a visit by a number of Kyrgyz muslims to the “Small Hajj” in Saudi Arabia; cases now stand at about 170,000 (August 2021), about 2.5% of the population.

Following WHO guidelines, laboratories tested nasal swab samples for SARS-CoV-2 using RT-PCR. Early in the outbreak, 13 laboratories for PCR diagnostics were opened in the country, including three mobile ones. Together with the local health, education and science ministries, international organizations, such as WHO, CDC, academic initiatives such as the Columbia University International Assistance Program (ICAP), and groups of foreign scientists, (from China and Russia), a series of training sessions on PPE, laboratory testing, treatment and follow-up were organized. This international cooperative approach, following the international efforts mentioned earlier, allowed the Kyrgyz authorities to be better prepared to deal with the pandemic.

Currently, research is being conducted on serological assessment of population immunity in seven regions of the republic and in the cities of Bishkek and Osh using different age groups. ELISA is used to test for the presence of SARS-CoV-2-specific IgA, IgM and IgG. Based on these results, national immunity to this coronavirus infection in the Kyrgyz Republic will be known and appropriate action taken. No vaccine development is underway in the country, since there are no suitable facilities available. However, China has recently gifted doses of the “Sinopharm” COVID vaccine and, despite some local resistance, about 8% of the population has had at least one dose.

## Crimean-Congo Hemorrhagic Fever in the Republic of Tajikistan

WHO prioritizes Crimean-Congo haemorrhagic fever virus (CCHFV) as one of seven epidemic-prone diseases: a “public health emergency” with an “urgent need for accelerated research” and is the most widespread tick-borne viral haemorrhagic fever infection in the world (14).

CCHF is notoriously difficult to diagnose, because early symptoms, including fever, myalgia, diarrhea, nausea, and vomiting, are often indistinguishable from those of more common tropical diseases (15). CCHF is endemic in Central Asia while the incidence and prevalence in countries such as Tajikistan is not yet widely understood.

In 2012, Public Health England (PHE)'s Virology and Pathogenesis group was invited to support the development of molecular CCHFV diagnostics in Tajikistan by its Ministry of Health. Accordingly, a cooperative programme was developed to implement a standard RT-PCR assay (16) at the Institute of Preventative Medicine (IPM) in the capital, Dushanbe (Figure 2). This included a series of training workshops in the UK and Tajikistan supported by the UK IBSP. Over a 2-year period, PCR diagnostics for CCHFV became a standard capability in the IPM laboratories. Collaboration, including the exchange of samples between the UK and Tajikistan, which supported the continued development of new RT-PCR assays, built capacity in country (17) and supported the UK's capability to rapidly detect imported CCHF and reduce onward transmission in the UK National Health Service. Based on the successful implementation of this laboratory diagnostic assay, the cooperative programme went on to work up the development of a field-capable nucleic acid test for CCHFV using novel isothermal Replicase Polymerase Amplification (RPA) chemistry (18). This new tool is ideally placed for use in-low resource settings and can monitor CCHF outbreaks at the point-of-need, such as in remote rural regions in affected countries. Its implementation in Tajikistan has also contributed to major new CCHF disease control programmes in the country. The UK's International Biological Security Program (IBSP), which is a global partnership with aims to strengthen national health systems; support research on vaccines, drugs and diagnostics, was also active in several locations including Kazakhstan.

As evidenced here and other parts of the world, the lack of a rapid, simple and affordable diagnostic in these early stages of disease is a serious problem, which leads to the propensity of the virus to cause nosocomial outbreaks where mortality rates of up to 80% have been reported (19–21). In other austere environments and regions, obtaining reagents and consumables for diagnostics can be difficult to obtain. In rural settings, the situation is exacerbated by limited health care facilities and initial spill-over events from wildlife tick vectors that go unrecognized until community outbreaks sustained by human-to-human transmission develop (22). Such a situation exists in Tajikistan which, in addition to having one of the highest national burdens of CCHF, also has the dubious distinction of occupying territory where CCHF was first described in the 11^th^ Century. Many severe cases have been recognized since the disease was first brought to modern medical attention over 60 years ago.

## Role of Science Networks: Formal and Informal

Scientific collaboration networks can exist formally or informally and can focus around any given specific disease topic, a technology such as genomics and sequencing, or an emerging field such as ecoimmunology. Formal networks are most likely funded and organized whereas informal networks are a subset of researchers that may be connected in some manner such as through institutions, professional societies and past collaborations. To address infectious diseases and biosurveillance, DTRA BTRP created the more formal Biological Threat Reduction Networks (BTRN) (23). As the name implies, BTRNs aim to connect scientists around the world with the shared mission of reducing biological threats. With the several existing cooperative biological engagement programs mentioned, the primary objectives include strengthening capabilities in detection and diagnostics and to have these scientific and technical capabilities become sustainable. One of the best ways of creating sustainable capabilities within countries is to connect researchers and the technical professionals together enabling cooperation or sharing expertise and information, as well as combating misinformation.

Informal networks are the connections, professional relationships, and source of contacts that scientists often leverage throughout their careers. These contacts include fellow researchers, peer colleagues, and mentor/mentees that connect at scientific conferences and related collaborations. These scientists maintain informal networks independently. As science becomes more multidisciplinary across disparate fields, the breadth of the informal networks between researchers is becoming larger and more diverse. For example, infectious disease research requires the understanding the ecology of an emerging or endemic infectious disease system where epidemiologists may work with meteorologists, sociologists, wildlife biologists, and geographers. With unlimited access between researchers through the internet, general connections are not endangered, however, trusted relationships and sustained connections are rarer. Cooperative engagement research is designed to build trusting and long-term relationships. The COVID-19 pandemic has led to an unprecedented amount of science and it was through the trusted collaborations that existed prior to the pandemic, that the critical initial data and information on the coronavirus was shared. Also, with the COVID-19 pandemic, most networks have had to move to become virtual networks. Having a low-cost virtual platform for connecting can help networks become sustainable into the future if and when funding ends.

During infectious disease outbreaks, both formal and informal networks are critical for a rapid and coordinated response. As it is often repeated, “if you exchange business cards on the first day of an outbreak, the pathogen has already won.” The return of investment for cooperative engagement programs (5) became evident immediately in the 2020 COVID-19 pandemic. Country partner researchers and diagnosticians quickly moved to detect and diagnose SARS-2 as it moved into and across regions. Both through formal and informal networks, researchers reached out to each other for advice on the specifics of PCR diagnostics, sequencing of the SARS-2, and general information on the behavior of the disease in humans. The COVID-19 pandemic has shown that the time and effort over the past 15 years in cooperative engagement paid off in the faster exchange of information, data, samples, and has overall built trust between scientists and countries. Cooperative engagement research designed to understand One Health systems has shown a high return on investment and has reduced the threat of global infectious disease spread (24).

## Conclusions

Continuing to operationalize cooperative research and infectious disease surveillance are essential complements to identify and mitigate the next outbreak. As illustrated in our three examples of cooperative biological research, prior efforts by agencies from many different countries have set up the scientific and medical communities in partner countries to deal rapidly and expertly with a biological threat outbreak. Global infectious disease outbreaks, such as the COVID-19 pandemic, require a global response. These agencies have primed personnel, with little prior skills, to recognize threats, deal with them and share techniques, data and ideas with colleagues across the world. Human and social factors such as trust building and political will influence the partnerships and networks. The activities described demonstrated the requisite trust needed to continue collaborations and avoid transactional one-off studies. Overall, the political will, which usually backs financial investment, has made COVID-19 easier to track, deal with clinically and attack through development of therapies and candidate vaccines. Sharing technology and ideas is only possible when partners are trained to understand their importance and carry out state-of-the-art techniques. Continuing these activities to enhance capabilities and capacities along with building trust will be required beyond the COVID-19 pandemic.

## Author Contributions

KY, FP, and KairT developed this concept along with JH, RH, JF, and SE. KaisT contributed content for the manuscript. All authors reviewed and agreed on the final submission.

## Conflict of Interest

FP was employed by the company EpiPointe, LLC. The remaining authors declare that the research was conducted in the absence of any commercial or financial relationships that could be construed as a potential conflict of interest.

## Publisher's Note

All claims expressed in this article are solely those of the authors and do not necessarily represent those of their affiliated organizations, or those of the publisher, the editors and the reviewers. Any product that may be evaluated in this article, or claim that may be made by its manufacturer, is not guaranteed or endorsed by the publisher.
